# Clinical Evaluation of Allergen Immunotherapy for Allergic Rhinitis

**DOI:** 10.3390/vaccines14040326

**Published:** 2026-04-07

**Authors:** Francesco Catamerò, Maria Chiara Bragato, Montserrat Alvaro Lozano, Giorgio Walter Canonica, Domingo Barber Hernández, Maria M. Escribese, Enrico Heffler, Oliver Pfaar, Umit Sahiner, Giovanni Paoletti, Mattia Giovannini

**Affiliations:** 1 Allergy Unit, Meyer Children’s Hospital IRCCS, 50139 Florence, Italy; mattia.giovannini@unifi.it; 2 Department of Health Sciences, University of Florence, 50139 Florence, Italy; 3 Personalized Medicine, Asthma and Allergy, IRCCS Humanitas Research Hospital, Rozzano, 20072 Milan, Italy; m.bragato4@campus.unimib.it (M.C.B.); canonica.gw@gmail.com (G.W.C.); heffler.enrico@gmail.com (E.H.); giovanni.paoletti@hunimed.eu (G.P.); 4 Pediatric Allergology and Clinical Immunology Department, Hospital Sant Joan de Déu, 08950 Barcelona, Spain; montserrat.alvaro@sjd.es; 5 Institut de Recerca Sant Joan de Déu, 08950 Barcelona, Spain; 6 Departament de Cirurgia i Especialitats Médico-Quirúrgiques, Universitat de Barcelona, 08950 Barcelona, Spain; 7 Department of Biomedical Sciences, Humanitas University, Pieve Emanuele, 20072 Milan, Italy; 8 Department of Basic Medical Sciences, Institute for Applied Molecular Medicine Nemesio Díez, School of Medicine, Universidad San Pablo-CEU, CEU Universities, Urbanización Montepríncipe, 28668 Boadilla del Monte, Spain; domingo.barberhernandez@ceu.es (D.B.H.); mariamarta.escribesealonso@ceu.es (M.M.E.); 9 RICORS “Red de Enfermedades Inflamatorias (REI)” (RD24/0007/0018), Instituto de Salud Carlos III, 28029 Madrid, Spain; 10 Section of Rhinology and Allergy, Department of Otorhinolaryngology, Head and Neck Surgery, University Hospital Marburg, Philipps-Universität Marburg, 35043 Marburg, Germany; oliver@pfaar.org; 11 Department of Pediatric Allergy, Faculty of Medicine, Hacettepe University, Ankara 06230, Türkiye; umsahner@yahoo.com

**Keywords:** allergen immunotherapy (AIT), efficacy, safety, clinical evaluation, respiratory allergy

## Abstract

**Background/Objectives**: Allergen immunotherapy (AIT), involving subcutaneous (SCIT) or sublingual (SLIT) administration of the culprit allergen, is the only treatment capable of modifying the natural course of allergic diseases, and provides lasting benefits in terms of symptom reduction and medication use. AIT for allergic rhinitis is acknowledged as safe and effective in both adults and children; however, no studies have comprehensively evaluated the safety and efficacy of AIT in these populations, integrating results from randomized controlled trials (RCTs) and real-world evidence (RWE). **Methods**: We evaluated data in the literature including studies from RCTs and RWE in which the safety and efficacy of AIT in both children and adults have been analyzed. A narrative literature search was conducted in PubMed up to January 2026 using the following keywords for the search string: “allergen immunotherapy,” “AIT,” “safety,” “efficacy,” “clinical outcome,” and “clinical evaluation.” **Results**: RCTs and meta-analyses showed that both SCIT and SLIT significantly reduced allergic symptoms and medication use and improved quality of life (QoL). Large SLIT tablet trials have confirmed its efficacy in adults and children, whereas RWE supports its effectiveness in broader populations. Safety data indicated that SCIT carries a small but higher risk of systemic reactions than SLIT, which mainly causes mild local effects. **Conclusions**: AIT was effective and safe for treating allergic rhinitis across RCT and RWE studies. Integrating RWE with RCT findings is essential for guideline development, particularly for capturing long-term outcomes and real-world applications.

## 1. Introduction

The burden of allergic diseases has grown substantially in recent years, currently impacting roughly one-quarter to one-third of adults and up to 40% of the pediatric population worldwide [1,2,3,4]. Allergic rhinitis (AR), whether occurring alone or with conjunctivitis (AR/C), can significantly disrupt sleep patterns, daily activities, and both academic and professional performance, and is commonly observed in conjunction with asthma [5,6,7,8,9].

Although minimizing exposure to relevant allergens is advisable, this approach is frequently difficult to implement, particularly for seasonal pollens [10]. Consequently, symptom-relieving medications remain the mainstay of management for many patients. While effective in controlling clinical manifestations, these treatments do not influence the underlying disease mechanisms or its long-term evolution and may be associated with unwanted effects [6,7,9,10,11].

In contrast, allergen immunotherapy (AIT), administered via subcutaneous (SCIT) or sublingual (SLIT) routes, represents the only available strategy capable of inducing disease modification on the natural history of the disease [10,12].

Evidence consistently demonstrates its ability to decrease symptom severity, enhance quality of life (QoL), and confer beneficial effects on asthma outcomes, with potential long-term disease-modifying properties, after a treatment duration of at least 3 years is generally required to achieve sustained long-term benefits [11,13,14,15,16,17].

Despite its demonstrated efficacy, the implementation of AIT in clinical practice remains limited. Contributing factors include economic considerations, concerns about safety, patient-related barriers, and difficulties in maintaining long-term adherence [18,19,20,21]. From a practical perspective, SCIT necessitates repeated healthcare visits, whereas SLIT requires sustained daily self-administration, both of which may negatively affect persistence and overall effectiveness [22,23,24]. Furthermore, access to AIT is uneven across Europe, with reimbursement policies varying widely and full financial coverage available in only a minority of countries [19,22,25].

AIT has a history spanning more than a century [26]. In 1911, Leonard Noon first demonstrated that repeated injections of grass pollen extract reduced allergic sensitivity to hay fever, with subsequent confirmation by Freeman of its clinical benefits during the pollen season [27,28]. In 1954, the first double-blind trial established the efficacy of subcutaneous grass pollen injections, which were later shown to act via high molecular weight proteins [29]. Over time, studies have demonstrated that the effects of AIT are allergen-specific and can provide long-term benefits, particularly after 3 years of continuous treatment [30]. Although SCIT administration has remained largely unchanged, the recognition of SLIT as a safe and effective alternative, supported by the World Health Organization (WHO) in 1998, marked an important milestone [31]. Since then, multiple randomized controlled trials (RCTs) have confirmed the safety, efficacy, and long-term disease-modifying effects of SCIT and SLIT [17,32,33,34,35,36,37,38].

Along with RCTs, AIT outcomes are also derived from complementary real-world evidence (RWE) studies, providing a comprehensive understanding of the benefits of AIT across various allergic conditions [19,39]. The Food and Drug Administration (FDA) defines RWE as “clinical evidence regarding the usage and potential benefits or risks of a medical product, derived from the analysis of real-world data (RWD) [40]. RWE can be generated by different study designs or analyses, including, but not limited to, RCTs (including large simple trials and pragmatic trials), and observational studies (prospective and/or retrospective) [41,42].

**Figure 1:**
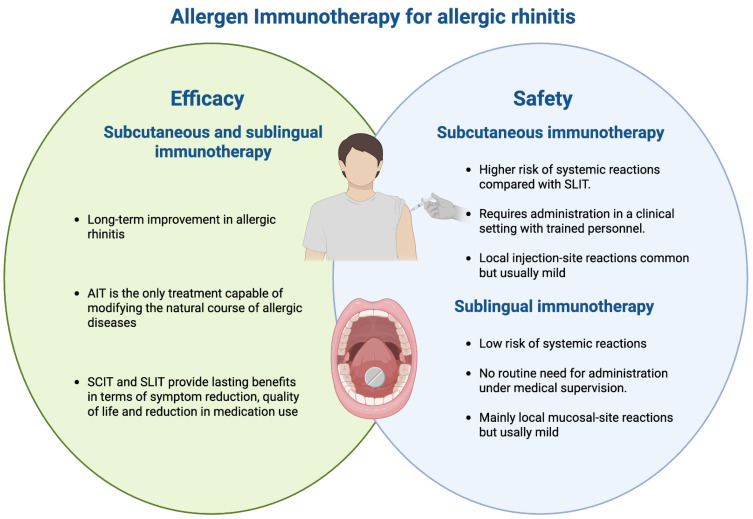
Efficacy and safety of allergen immunotherapy in allergic rhinitis. AIT, allergen immunotherapy; SCIT, subcutaneous immunotherapy; SLIT, sublingual immunotherapy.

The purpose of this narrative review was to examine the available literature by integrating findings from both RCTs and RWE to provide an updated and balanced overview of the clinical evaluation of AIT for rhinitis across different age groups. A narrative literature search was conducted in PubMed up to January 2026 using the following keywords: “allergen immunotherapy,” “AIT,” “safety,”, “efficacy”, “clinical outcome” and “clinical evaluation”. Articles were selected based on their relevance to the clinical use of AIT in AR with or without allergic asthma, including studies evaluating the efficacy, safety, adherence, and long-term outcomes. While priority was given to RCTs, meta-analyses, and large observational real-world studies, additional relevant reviews and guideline documents were also considered to contextualize the evidence (Figure 1).

## 2. Allergen Immunotherapy Efficacy

When referring to AIT efficacy, it is crucial to differentiate between two terms: “efficacy” and “effectiveness.” Efficacy is a broader term indicating whether an intervention provides more benefits than damages, while effectiveness indicates whether an intervention provides benefits under the usual circumstances of healthcare practice [43,44].

Evaluating AIT efficacy can be challenging for two main reasons:

1. The wide variety of AIT product compositions (efficacy must be demonstrated for every product with different compositions rather than for a class) [45,46].
2. AIT studies are seldom comparable due to the diversity of allergen extracts, doses, and dosing regimens. In addition, study designs, inclusion criteria, and outcome assessments often differ [43].

The clinical efficacy of AIT is predominantly assessed using patient-reported outcomes (PROs), with combined symptom and medication scores (CSMS), such as those standardized by the EAACI [47] or total combined symptoms, serving as primary endpoints [48,49,50,51,52,53]. These composite scores capture daily symptom severity, including rhinorrhea, nasal itching, sneezing, congestion, and ocular symptoms, and quantify the use of rescue medications such as oral antihistamines, intranasal corticosteroids, and decongestants [48,50,51,54]. This approach provides the greatest effect size and reflects both the disease burden and treatment impact, although a universally accepted scoring system remains lacking. Secondary endpoints commonly include health-related QoL measures such as the Rhinoconjunctivitis Quality-of-Life Questionnaire (RQLQ) or Asthma Control Test (ACT), individual symptom scores, visual analog scales (VAS), and frequency of symptom-free days [7,9,11,55,56,57].

In vivo biomarkers, including nasal, conjunctival, and bronchial provocation tests or allergen exposure chambers [58], are valuable for diagnostic and dose-finding purposes and for assessing reductions in skin test reactivity. However, in vitro biomarkers (e.g., specific IgE, IgG4, and T-cell responses) remain largely investigational and have not yet been validated as reliable predictors of clinical efficacy [50,51,59,60,61,62,63].

Nonetheless, both SCIT and SLIT have demonstrated significant improvements in allergic respiratory symptoms.

### 2.1. Subcutaneous Immunotherapy Efficacy

SCIT has a long history as an allergy treatment and is known for its ability to modulate underlying immunological processes [27,64]. Mechanistically, SCIT enhances the synthesis of allergen-specific IgG (“blocking” antibodies) while decreasing allergen-specific IgE, and reduces allergen-specific inflammation [64,65]. Meta-analyses of RCTs have consistently shown that SCIT significantly reduces allergic symptoms and medication use for AR/C [66]. It has been shown to modify the underlying pathological mechanisms, offering sustained long-term efficacy in AR/C even after treatment cessation, and may prevent the development of asthma [49]. Systematic reviews and meta-analyses, such as those by Abramson et al. (2003) and Dhami et al. (2017), have confirmed the efficacy of SCIT, with demonstrated reductions in asthma symptoms and medication scores, and improved asthma-specific QoL [67,68].

A recent double-blind placebo-controlled trial (DBPCT) in which SCIT was performed with a glutaraldehyde-modified house dust mite (HDM) extract comprising a 1:1 mixture of *D. pteronyssinus* and *D. farinae* indicated that SCIT was effective in the subgroup of patients with moderate-to-severe disease and highlighted the importance of possible modified allergen extract usage [69].

Notably, a randomized, placebo-controlled trial (MITAR study) investigating 1-year HDM SCIT in patients with moderate-to-severe AR/C using an intranasal corticosteroid (ICS) at baseline found that SCIT significantly reduced symptoms and medication use [49]; however, the changes were not significantly different from those in the placebo group, which also received an ICS at baseline. This finding contrasts with those of earlier DBPCTs, in which the placebo arm typically received rescue medication or followed a stepwise regimen.

RCTs have further supported the effectiveness of SCIT in patients with AR/C and allergic asthma, showing reductions in symptom scores of 20% to 36.5% compared to placebo [70,71,72]. While some meta-analyses found no significant effect on forced expiratory volume in 1 s (FEV1), a modest increase in expiratory flow and a decrease in allergen-specific airway hyperreactivity were observed [66,67,73,74,75,76]. A meta-analysis of pediatric asthma found no significant difference in Total Asthma Symptoms Score or FEV1 improvement between SLIT and SCIT, although SLIT showed higher asthma improvement rates [75]. SCIT has also shown long-term benefits, with 3 years of continuous treatment providing sustained responses for up to 3 years after discontinuation [77,78].

The RWE for SCIT provides crucial insights into its broader effectiveness. The REACT study, the largest and most comprehensive AIT effectiveness study to date, confirmed the long-term and sustained effectiveness of AIT (SCIT or SLIT) over a 9-year follow-up period [79,80]. It demonstrated sustained reductions in AR/C and asthma medication use, improved asthma control, prevention of asthma exacerbations, and a reduced likelihood of pneumonia and hospitalization in AIT-treated patients [79,80]. The study also highlighted that these beneficial effects on asthma were found in a less severe asthmatic population (only 4% at treatment step 4), suggesting that AIT may prevent the progression from mild to more severe asthma and should be considered earlier [80]. A South American pediatric real-life prospective study assessed the effectiveness and safety of glutaraldehyde-polymerized HDM extract SCIT in children (3–11 years old) with AR/C with or without asthma [81]. Over 1 year, there was a significant 50% reduction in the CSMS for AR/C, with 78% of patients achieving at least a 20% reduction [81]. The ACT scores also significantly improved, and asthma medication use significantly decreased after 1 year. This finding supports the effectiveness and safety of adjuvanted SCIT in real-world pediatric settings [81,82]. Finally, other RWE studies conducted in both adults and children reinforced the early, sustained, and long-term effectiveness of SCIT in hampering AR/C, asthma progression, and asthma onset [83,84,85,86,87].

### 2.2. Sublingual Immunotherapy Efficacy

SLIT includes two main pharmaceutical formulations: liquid drops and rapidly dissolving tablets, administered under the tongue. Although SLIT drops have been widely used in clinical practice in several countries for many years, their formulations are often heterogeneous, including mixtures of allergens, with many lacking robust evidence from large RCTS. In contrast, SLIT tablets are standardized products that have undergone extensive clinical development programs and obtained regulatory approval in multiple regions, including Europe and North America.

Therefore, while acknowledging the broader clinical use of SLIT drops worldwide, the present review primarily focuses on evidence-based SLIT tablet formulations supported by high-quality clinical trials and regulatory evaluations.

The efficacy of SLIT, particularly in its standardized tablet form, has been well-demonstrated in numerous RCTs. Although its mechanisms are generally parallel to those of SCIT, studies suggest that IgA antibodies are the dominant isotype following SLIT, in contrast to the high levels of IgG generated by SCIT [65].

Approved SLIT tablets for allergens such as grass, ragweed, HDM, birch tree pollen, and Japanese cedar have consistently shown significant clinical efficacy in large DBPCTs [72,88,89,90,91,92,93,94,95,96,97,98]. For AR/C, these trials frequently report 30–40% improvement over placebo in combined symptom and medication scores for seasonal allergies and 15–20% improvement for perennial HDM allergy [72]. For instance, a 24-week North American study of timothy grass SLIT-tablet involving 1501 patients demonstrated a 23% improvement in combined symptom and medication scores over the entire pollen season and a 29% improvement during the peak season, compared to placebo (*p* < 0.001), along with a 17% improvement in health-related QoL (*p* = 0.02) [88,89,92]. A five-grass pollen SLIT tablet study in North America showed a 28% improvement [93]. Ragweed SLIT tablet trials have shown 11–23% improvement in combined symptom and medication scores during the peak season, with effects in both mono- and polysensitized individuals [99,100]. For HDM SLIT tablets, a North American field study reported a 17% improvement in total combined rhinitis symptoms and medication scores, with comparable efficacy observed across age groups, sex, ethnicity, and baseline asthma status [88]. European studies on HDM SLIT tablets also showed an 18% improvement in rhinitis scores and noted that treatment could reduce symptoms during other pollen seasons in polysensitized patients, suggesting a broader impact beyond the primary allergen by reducing T-helper type 2 priming [101]. Birch tree pollen SLIT tablets reduced symptoms during the cross-reactive hazel-alder pollen seasons (*p* < 0.0001) [102].

Recently, an increasing number of successful SLIT trials have been reported in the pediatric population [37,38]. Evidence from pivotal phase III trials in the pediatric population has demonstrated that SLIT tablets provide clinically meaningful and statistically robust benefits in children with moderate to severe AR/C. Large, randomized, DBPCTs in children aged 5–17 years with tree pollen allergy and those aged 5–11 years with HDM-induced disease showed consistent reductions of approximately 20–22% in combined symptom and medication scores versus placebo, alongside significant improvements in disease-specific QoL despite free access to rescue medication. Treatment effects were evident across subgroups, including in children with and without asthma, and were supported by immunological markers of tolerance (increased allergen-specific IgG4 levels and blocking activity) [37,38].

Compared to pharmacotherapy, SLIT tablets generally match or exceed symptom relief, with some studies showing greater relative effects than montelukast or oral antihistamines and comparable efficacy to intranasal corticosteroids for seasonal AR/C [96]. For patients with AR/C and allergic asthma, seasonal allergen SLIT trials typically include asthma outcomes as secondary endpoints; however, HDM SLIT tablets have demonstrated significant benefits in RCTs. A pivotal trial by Virchow et al. showed an asthma exacerbation risk reduction of ≥30% with HDM SLIT-tablet and a significant 36% relative risk reduction in moderate-severe asthma exacerbations [103]. A study by Mosbech et al. found significant reductions in ICS use with HDM SLIT tablets while maintaining asthma control, demonstrating a significant difference compared to placebo (*p* = 0.004) [104]. These findings led to the inclusion of HDM AIT in the GINA report on asthma treatment [105].

The RWE for SLIT complements these RCT findings by demonstrating its effectiveness under routine healthcare conditions. As highlighted for SCIT, the REACT study confirmed the long-term and sustained effectiveness of AIT, including SLIT, over 9 years of follow-up [80]. Subgroup analyses of the REACT study found that SCIT and SLIT tablets showed similarly greater reductions in AR/C prescriptions than the controls. Specifically, the SQ grass SLIT tablet demonstrated sustained reductions in AR prescriptions for up to 7 years [33]. These findings suggest that SLIT can prevent AR progression and impede the development of asthma (Table 1).

Moreover, early initiation of SLIT tablets in childhood provides important long-term clinical and economic benefits compared to later treatment. Evidence from the Benefits of Early Allergen Immunotherapy Initiation (BEAT) model and the Grazax Asthma Prevention (GAP) trial showed that starting SLIT at a younger age significantly reduced the risk of progression from AR to asthma [106,107]. Initiating treatment at 5 years of age decreased the risk of developing asthma by approximately 34% compared with starting treatment at 12 years. Long-term projections indicated that approximately 19% of children treated at 5 years developed asthma over 20 years, compared with 24% in whom treatment began at 7 years and 29% in whom treatment began at 12 years. In addition, SLIT demonstrated a disease-modifying effect, with sustained reductions in AR/C symptoms of approximately 22–30% during treatment and follow-up. From a health-economic perspective, earlier SLIT initiation is associated with lower long-term healthcare costs, owing to reduced asthma incidence and better disease control. Over a 20-year period, starting treatment at the age of 5 years resulted in lower cumulative costs compared to initiation at older ages.

For SLIT drops, while regulatory approval is limited in the U.S. and Canada owing to high heterogeneity in studies, a product-line meta-analysis focused on the Index of Reactivity (IR) of SLIT liquid formulations for AR/C demonstrated significant efficacy. This meta-analysis, based on 25 RCTs, found that IR-SLIT liquid was significantly more effective than placebo at reducing both symptoms and medication scores (*p* < 0.0001) [108]. The RWE from the EfficAPSI study also supports the effect of liquid SLIT on the onset and progression of asthma [109].

**Table 1:** Main real-world studies on the effectiveness of SCIT and SLIT.

Authors	Year	Population	Results
Milani et al. EFESO trial [110]	2008	154 patients with allergic rhinitis + 151 controls	SLIT treatment in AR is associated with better quality of life, diminished use of symptomatic drugs, and lower incidence of asthma and new sensitizations.
Zielen et al. [85]	2018	2851 patients sensitized for grass pollen + 71,275 control patients	SLIT tablets for grass pollen decreased the use of symptomatic drugs for AR/C and of asthma medication with less frequent asthma exacerbation
Wahn et al. [86]	2019	9001 patients sensitized for birch pollen + 45,005 control patients	Reduced AR/C and asthma medications, less frequent asthma exacerbation
Devillier et al. [87]	2019	1099 grass pollen SLIT patients + 27,475 control patients	Prescription of grass pollen SLIT tablets reduced the dispensing of AR and asthma medications
Jutel et al. [111]	2020	2350 patients sensitized to house dust mite + 64,740 controls	Reduced AR/C and asthma medications, reduced asthma development
Vogelberg et al. [84]	2020	11,931 SCIT patients and 10,698 SLIT patients sensitized for grass and tree pollen	SCIT and SLIT have been proven to ameliorate symptom scores, medication scores, and combinations of both in patients with AR/C and asthma.
Fritzsching et al. REACT study [80]	2021	46,024 patients with AR/C and with (14,614) or without asthma + 46,024 controls	Reduction in symptomatic drug usage, greater likelihood of stepping down asthma treatment, reduction in asthma exacerbation and pneumonia with antibiotic prescription
Pavon-Romero et al. [112].	2021	786 clinical records with SCIT from 2005 to 2018, comparing the clinical characteristics of patients with ARs versus a group of a similar number of patients who completed SCIT without reactions	Most reactions were mild (grade 1), with fewer grade 2 reactions and no grade 3, grade 4, or 5 (fatality). SCIT demonstrated a favorable safety profile in pediatric patients, with infrequent and predominantly mild ARs.
Cardona-Villa et al. [81]	2025	49 children diagnosed with HDM-AR/C, with or without asthma, who were treated with SCIT for 1 year.	Reduction in the CSMS for AR/C. ACT scores also significantly improved, and asthma medication use significantly decreased after 1 year.
Demoly et al. (EfficAPSI study) [109]	2024	112,492 AR patients treated with personalized liquid SLIT vs 333,082 matched controls without AIT; patients with and without pre-existing asthma, long-term follow-up (median ~7–8 years)	SLIT-liquid exposure was associated with a significant reduction in asthma onset and progression: ~35–40% lower risk of new asthma events across sensitive, specific and combined definitions; ~one-third reduction in GINA treatment step-up in patients with pre-existing asthma and increased likelihood of step-down.
Mosges et al. [113]	2025	101 patients with cat allergy (91 treated with depigmented-polymerized cat allergoid), adolescents and adults, during SCIT up-dosing phase under real-world conditions	SCIT with depigmented-polymerized cat allergoid was safe and well tolerated: ~50% reported ADRs, mainly delayed local reactions; systemic ADRs were rare and non-persistent. ADR incidence did not differ by age group or up-dosing regimen.

## 3. Allergen Immunotherapy Safety

AIT is widely considered safe and well-tolerated, especially when delivered under appropriate medical supervision. Although both SCIT and SLIT are effective disease-modifying interventions, their safety profiles differ in terms of the incidence, type, and severity of adverse reactions (ARs). Understanding these differences is critical for optimizing patient selection, monitoring, and counseling.

### 3.1. Subcutaneous Immunotherapy Safety

SCIT is associated with a higher risk of systemic allergic reactions (SARs), including anaphylaxis, than SLIT; therefore, it must be administered under strict medical supervision in settings equipped for the prompt management of anaphylaxis [31,59,114]. Patients are typically monitored for at least 30 min following each injection [115,116]. In conventional SCIT regimens, the occurrence of SARs, the most clinically significant ARs of AIT, is approximately 0.2% (range 0.01–0.3%) [43,117]. Surveillance studies have reported that SARs graded 3–4 according to the World Allergy Organization criteria (Table 2) occur in approximately 2% of patients, with two-thirds of these events observed in individuals with pre-existing asthma [118,119,120]. For safety reasons, patients with severe and uncontrolled asthma (FEV_1_ or peak flow <70% of predicted) are generally excluded from SCIT clinical trials [10,121]. Fatal events remain exceedingly rare; U.S. surveillance data from 2008 to 2016 documented approximately 1 fatality per 9 million injections, although near-fatal reactions have been reported at a rate of 5.4 per million injections [122,123,124]. Uncontrolled asthma has consistently been identified as the strongest predictor of severe and near-fatal reactions [49,122,123,124].

The most common ARs during SCIT are local reactions (LRs) at the injection site, including itching, swelling, and redness, which typically occur within 30 min [115,116,125]. Their frequencies may be underestimated because many events occur outside the physician’s office [126]. The composition of SCIT products also influences the risk, with clinical trials indicating that allergoid-based formulations are associated with fewer ARs than natural extracts [127,128,129].

The RWE supports these findings. The prospective multicenter Allergen Immunotherapy Adverse Events Registry (ADER), which included 755 SCIT courses in adults, confirmed that both the subcutaneous route and presence of asthma are major risk factors for ARs. ADER also demonstrated that natural extracts were more likely to induce reactions than allergoids and identified an increased risk from birch and mugwort pollen extracts [121]. Overall, the ADER concluded that SCIT is generally safe for adults with respiratory allergies, with most ARs being mild and severe events remaining rare.

Modified allergens used in SCIT have been developed to improve safety by reducing the IgE-binding capacity while preserving immunogenicity and the ability to induce long-term immune tolerance. Among these, allergoids demonstrated a very favorable safety profile. Polymerized allergoids used in SCIT show extremely low rates of SRs, estimated at 0.0005–0.01% per injection [130]. In addition, trials using high-dose grass pollen polymerized allergoids (e.g., aluminum-adsorbed preparations) have shown that intra-seasonal initiation of therapy does not increase SRs while still inducing robust immunological responses, such as increased allergen-specific IgG4 [131]. Other engineered approaches, including recombinant allergens, peptides, and allergen–carrier fusion proteins, have demonstrated good safety profiles in clinical trials. For example, SCIT with recombinant Timothy grass allergens was well tolerated even at doses of up to 120 μg without major adverse effects, while peptide-based strategies and epitope-targeted vaccines aim to further minimize IgE-mediated reactions [132].

**Table 2:** World Allergy Organization subcutaneous and sublingual immunotherapy systemic reaction grading system [133].

Grade 1	Symptom(s)/sign(s) of one organ system present: cutaneous, upper respiratory, conjunctival, nausea
Grade 2	Symptom(s)/sign(s) of more than one organ system present OR lower respiratory symptom OR gastrointestinal symptoms OR uterine cramps
Grade 3	Lower respiratory symptoms more severe than grade 2 (≥40% PEF or FEV1 drop, not responding to inhaled bronchodilator) OR laryngeal, uvula, or tongue edema
Grade 4	Respiratory failure OR Hypotension
Grade 5	Death

### 3.2. Sublingual Immunotherapy Safety

SLIT is generally regarded as having a more favorable safety and tolerability profile than SCIT, offering a superior risk–benefit ratio largely because of fewer SARs, with no fatalities reported in clinical trials for SLIT tablets [43,59,117,119,134,135,136]. Its safety profile permits at-home administration after the first dose is administered under medical supervision [102]. The main ARs are LRs within the oral cavity—such as oral pruritus, throat irritation, and tongue edema. These reactions are typically mild-to-moderate in intensity, transient (often resolving within 30 min post-administration and usually within 2–4 weeks of starting treatment), and rarely lead to treatment discontinuation (in approximately 5% of patients) [137,138,139].

For SLIT tablets, SARs are rare, with a large clinical trial database indicating an anaphylactic rate of 0.02% [140,141,142]. US clinical trials of SLIT tablets have reported few SARs, and no serious events or airway compromise [143]. Non-US studies have reported an SAR rate of 0.056% [140].

The FDA requires an accompanying prescription for a self-injectable epinephrine autoinjector for SLIT tablets in the US; however, this is not a universal requirement in other countries.

A history of eosinophilic esophagitis (EoE) is a contraindication for SLIT tablets, although a definitive causal link between SLIT tablets and EoE has yet to be established [80,143].

RWE reinforces this favorable safety profile. The REACT study reported one anaphylactic event in 3754 subjects treated with SLIT tablets. Similarly, the ADER registry, which evaluated 1060 SLIT courses, found that ARs were predominantly mild and severe events were rare. Overall, the extensive RWE confirmed that SLIT is generally safe and well-tolerated in adults and children with respiratory allergies.

## 4. Conclusions

AIT, delivered as SCIT or SLIT, remains the only established disease-modifying intervention for respiratory allergies to date, with convergent evidence from RCTs and RWE demonstrating consistent reductions in symptoms and medication use, along with improvements in QoL. Long-term follow-up data indicate that these benefits, if the treatment is maintained for at least 3 years, may persist beyond treatment discontinuation, suggesting sustained effects on disease progression.

SCIT shows robust efficacy across AR/C but necessitates administration under medical supervision due to a higher, albeit infrequent, risk of SARs. SLIT provides efficacy while offering a more favorable safety profile dominated by transient LRs and a lower occurrence of SARs.

Large RWE cohorts further support the effectiveness and safety of both SCIT and SLIT in routine clinical practice, broadening the evidence base to include diverse patient populations. Collectively, these findings underscore the role of AIT as a core element of respiratory allergy management. As there is no generic class effect on AIT efficacy and safety, treatment decisions should rely on product-specific evidence derived from clinical trials and real-world studies, in accordance with current guideline recommendations. Importantly, comparisons between SCIT and SLIT should not be interpreted as evidence of route-based superiority but rather as reflections of differences among individual products, study designs, and clinical contexts.

Adherence and persistence are the key determinants of AIT effectiveness. Administration models differ substantially between SCIT, which is delivered under medical supervision in clinical settings, and SLIT, which is typically self-administered at home. While supervised administration may facilitate adherence monitoring and allow the direct verification of treatment persistence, home-based regimens rely more heavily on patient engagement, education, and long-term motivation. Real-world studies suggest that adherence to AIT can decline over time, particularly given the recommended multi-year treatment duration required to achieve sustained clinical benefits. These differences highlight the importance of structured follow-up strategies, digital adherence tools, and patient-centered education programs to support persistence in therapy. Therefore, optimizing adherence to routine practice is critical for translating the efficacy observed in clinical trials to long-term effectiveness in real-world populations.

Integrating RWE with RCT data is essential to inform future guideline development, particularly for capturing long-term effects and real-world applicability.

## Data Availability

No new data were created or analyzed in this study. Data sharing is not applicable to this article.

## Abbreviations

The following abbreviations are used in this manuscript:

AIT	allergen immunotherapy
AR	allergic rhinitis
AR/C	allergic rhinitis with or without conjunctivitis
SLIT	sublingual immunotherapy
SCIT	subcutaneous immunotherapy
QoL	quality of life
RCTs	randomized controlled trials
RWE	real-world evidence
RWD	real-world data
PROs	patient-reported outcomes
CSMS	combined symptom and medication scores
RQLQ	Rhinoconjunctivitis Quality-of-Life Questionnaire
ACT	Asthma Control Test
VAS	visual analogue scales
FEV1	forced expiratory volume in one second
HDM	house dust mite
ADER	Allergen Immunotherapy Adverse Events Registry
FDA	Food and Drug Administration
WHO	World Health Organization
ICS	intranasal corticosteroid
DBPCTs	double-blind placebo-controlled trials
ARs	adverse reactions
SARs	systemic allergic reactions
LRs	local reactions
